# Total and Differential Leukocyte Counts in Relation to Incidence of Diabetes Mellitus: A Prospective Population-Based Cohort Study

**DOI:** 10.1371/journal.pone.0148963

**Published:** 2016-02-18

**Authors:** Yan Borné, J. Gustav Smith, Peter M. Nilsson, Olle Melander, Bo Hedblad, Gunnar Engström

**Affiliations:** 1 Department of Clinical Sciences, Malmö, Lund University, Malmö, Sweden; 2 Department of Cardiology, Clinical Sciences, Lund University and Skåne University Hospital, Lund, Sweden; 3 Center for Human Genetic Research, Massachusetts General Hospital and Harvard Medical School, Boston, Massachusetts, United States of America; 4 Cardiovascular Research Center, Massachusetts General Hospital and Harvard Medical School, Boston, Massachusetts, United States of America; 5 Program of Medical and Population Genetics, Broad Institute of MIT and Harvard, Cambridge, Massachusetts, United States of America; 6 Department of Internal Medicine, Skåne University Hospital, Malmö, Sweden; Tulane School of Public Health and Tropical Medicine, UNITED STATES

## Abstract

**Objective:**

High concentrations of leukocytes in blood have been associated with diabetes mellitus. This prospective study aimed to explore whether total and differential leukocyte counts are associated with incidence of diabetes. A missense variant R262W in the *SH2B3* (SH2B adaptor protein 3) gene, coding for a protein that negatively regulates hematopoietic cell proliferation, was also studied in relation to incidence of diabetes.

**Methods and Results:**

Leukocyte count and its subtypes (neutrophils, lymphocytes and mixed cells) were analyzed in 26,667 men and women, 45–73 years old, from the population-based Malmö Diet and Cancer study. Information about the R262W polymorphism (rs3184504) in *SH2B3* was genotyped in 24,489 subjects. Incidence of diabetes was studied during a mean follow-up of 14 years. Cox proportional hazards regression was used to examine incidence of diabetes by total and differential leukocyte counts. Mendelian randomization analysis using R262W as an instrumental variable was performed with two-stage least squares regression. A total of 2,946 subjects developed diabetes during the follow-up period. After taking several possible confounders into account, concentrations of total leukocyte count, neutrophils and lymphocytes were all significantly associated with incidence of diabetes. The adjusted hazard ratios (95% confidence interval; quartile 4 vs quartile 1) were 1.37 (1.22–1.53) for total leukocytes, 1.33 (1.19–1.49) for neutrophils and 1.29 (1.15–1.44) for lymphocytes. The R262W polymorphism was strongly associated with leukocytes (0.11x10^9^ cells/l per T allele, *p* = 1.14 x10^-12^), lymphocytes (p = 4.3 x10^-16^), neutrophils (p = 8.0 x10^-6^) and mixed cells (p = 3.0 x10^-6^). However, there was no significant association between R262W and fasting glucose, HbA_1c_ or incidence of diabetes.

**Conclusions:**

Concentrations of total leukocytes, neutrophils and lymphocytes are associated with incidence of diabetes. However, the lack of association with the R262W polymorphism suggests that the associations may not be causal, although limitations in statistical power and balancing pleiotropic effects cannot be excluded.

## Introduction

Inflammation has been repeatedly associated with diabetes mellitus, both in cross-sectional and prospective studies [1–5]. A meta-analysis of 20 prospective cohorts and cross-sectional studies found that raised leukocyte concentrations are associated with higher risk of type 2 diabetes [3]. However, results were inconsistent across studies [2–4, 6, 7], and the authors pointed out that results from the meta-analysis likely represent an overestimate due to publication bias and inability to control for potential confounders in all studies [3]. Most studies are cross-sectional and few prospective studies have examined the role of leukocytes in the development of diabetes. The Atherosclerosis Risk in Communities (ARIC) study from the USA found elevated leukocyte count to be associated with development of diabetes among 12,330 non-diabetic individuals aged 45–64 years after a mean follow-up of 7 years [2]. The National Health and Nutrition Examination Survey Epidemiologic Follow-up Study (NHEFS) found leukocyte count to be significantly and positively related to diabetes incidence with a dose-response relationship [6]. A high leukocyte count was found to predict impaired glucose tolerance and type 2 diabetes in Pima Indians [4]. In the Cardiovascular Health Study (CHS), only C-reactive protein (CRP) and not leukocyte count was associated with the development of diabetes [7].

Since gene variants are inherited and usually not subject to confounding [8], it is possible to use genetic polymorphisms as instrumental variables (IV) to explore the relationship between a trait and a certain outcome, without confounding bias from measured and unmeasured risk factors. We used a missense polymorphism (R262W, SNP rs3184504) in the SH2B adaptor protein 3 (*SH2B3)* gene to examine the effect of leukocyte count on diabetes. The R262W polymorphism is known to be associated with increased concentrations of leukocytes and its sub-populations [9, 10]. The SH2B3 gene codes for a protein that negatively regulates the hematopoiesis in knock-out models [11]. Loss of function of SH2B3 has been identified as a risk factor for leukemia [12].

The aim of this prospective study was to explore the relationship between total and differential leukocyte count and incidence of diabetes in an urban population.

## Subjects and Methods

### Study population

The population-based cohort Malmö Diet and Cancer study (MDC), from the city of Malmö in southern Sweden, was used in this study [13, 14]. In brief, all women born between 1923 and 1950 and men born between 1923 and 1945 living in Malmö city were invited to the MDC study during the period March 1991 to September 1996. A total of 30 447 individuals participated in clinical examinations at the screening center and filled in a self-administered questionnaire out of an eligible population of ≈74 000 individuals http://atvb.ahajournals.org/content/32/2/533.long-ref-17#ref-17. DNA was available for 28 767 subjects.

Subjects with history of diabetes (n = 1 311) at the baseline examination were excluded. In order to exclude individuals with severe inflammation, analyses were restricted to participants with information on total leukocyte counts less than 20.0 × 10^9^ cells/L [15]. In addition, 888 participants without complete information on covariates were excluded. Thus, the final study population in the project consisted of 26 667 subjects (10 364 men (38.9%) and 16 303 women (61.1%), aged 45–73 years. A random subsample from the MDC cohort, the MDC cardiovascular cohort (MDC-CV, n = 6,103), was invited to take part in a study of the epidemiology of carotid artery disease between October 1991 and February 1994 [16]. The additional examinations in this sub-cohort included measurements of fasting whole blood glucose, hemoglobin A1c (HbA_1c_) and CRP.

The ethics committee at Lund University Lund, Sweden, approved the study (LU 51/90) and all participants provided informed written consent.

### Baseline examinations

The examinations were performed by trained nurses at the screening center. Blood pressure (mmHg) was measured using a mercury-column sphygmomanometer after 10 minutes of rest in the supine position. Standing height (m) was measured with a fixed stadiometer calibrated in centimeters. Weight was measured to the nearest 0.1 kg using balance-beam scale with subjects wearing light clothing and no shoes. Body mass index (BMI) was calculated as weight (kg) divided by the square of the height (m^2^). Waist was measured as the circumference (cm) between the lowest rib margin and iliac crest.

Information on family history of diabetes, current use of lipid-lowering, blood pressure-lowering or anti-diabetic medications, smoking habits, leisure-time physical activity, education level and marital status were obtained from a self-administered questionnaire [13]. Family history of diabetes was defined as known diabetes in at least one first-degree relative [17]. History of myocardial infarction or stroke at the baseline examination was retrieved from the Swedish Hospital Discharge Register and the Stroke register in Malmö [18, 19]. Subjects were categorized into current smokers (i.e. those who smoked regularly or occasionally) or non-smokers (i.e. former smokers and never smokers). Low level of leisure-time physical activity was defined as the lowest quartile of a score revealed through 18 questions covering a range of activities in the 4 seasons. The evaluation of the questionnaire has been previously reported [20]. Educational level was divided into three groups: school year <9, 9–12 and > 12, respectively [21]. Marital status was categorized into married or not.

### Laboratory measurements

Total and differential leukocyte counts (neutrophils, lymphocytes, and a group of mixed cell types including monocytes, eosinophils and basophils) were counted in heparinized blood samples using a SYSMEX K1000 automatic counter (Sysmex Europe, Norderstedt, Germany). The analyses were performed consecutively at the time of the screening examination, at the central laboratory of Malmö University Hospital.

HbA1c and whole blood glucose was measured according to standard procedures at the Department of Clinical Chemistry. HbA_1c_ was measured by ion exchange chromatography, with reference values of 3.9–5.3% in non-diabetic individuals. Insulin was measured by a radioimmunoassay in mIU/ L and the HOMA index was calculated as fasting insulin*glucose/22.5 [16]. CRP was analyzed using a high-sensitive assay, Tina-quant^®^ CRP latex assay (Roche Diagnostics, Basel, Switzerland).

#### Incidence of diabetes

All subjects were followed from the baseline examination until first diagnosis of diabetes, death, emigration from Sweden or December 31^st^, 2009, whichever came first. Cases of new-onset diabetes in the MDC cohort were identified from several sources [22, 23]. In short, incident diabetes was identified from the Malmö HbA_1c_ register (MHR) (56% of all cases), the Swedish National Diabetes Register (NDR) (14%), the Swedish inpatient register (40%), the Swedish outpatient register (38%), the nationwide Swedish drug prescription register (65%) and the regional Diabetes 2000 register of the Skåne region (22%) [22, 23]. In addition, 44% of cases were identified at re-examinations of the cohort [24]. At least two independent sources confirmed the diagnosis for 71.6% of the cases, and 53% of cases were identified in three independent data sources. NDR and the Diabetes 2000 register required a physician´s diagnosis according to established diagnostic criteria (fasting plasma glucose concentration of > = 7.0 mmol/L, which corresponds to a fasting whole blood glucose of > = 6.1 mmol/L, measured on 2 different occasions). The MHR at the Department of Clinical Chemistry, Malmö University Hospital, analyzed and recorded all HbA_1c_ samples taken in institutional and non-institutional care in the greater Malmö area from 1988 onwards. Individuals who had at least two HbA_1c_ recordings > = 6.0% in the MHR with the Swedish Mono-S standardization system (corresponding to 7.0% according to the US National Glycohemoglobin Standardization Program) after the baseline examination were defined as incident diabetes cases.

### Polymorphism in the *SH2B3* gene

The missense polymorphism R262W (rs3184504), which previously has been associated with concentrations of leukocytes, neutrophils and lymphocytes [9, 10] was genotyped in 24,489 subjects. Based on results from previous GWAS studies of leukocyte count in subjects with European ancestry [25–27], we also tested whether polymorphisms in the 17q21 locus (rs4065321, rs3859192, rs9916158, rs4794822 and rs17609240) were useful as instrumental variables in this study. These genotypes were studied in relation to leukocyte count in a subsample of 4600 individuals, but none of the SNPs from the 17q21 locus reached p<0.5 x10^-8^ for the association with leukocyte count. Since we also found a significant inverse relationship between insulin resistance and the 17q21 locus, both in the literature [28] and in our data, we decided to use the R262W polymorphism only.

DNA was extracted from peripheral blood cells and assigned to batches without regard to disease status or personal identity. Batches were genotyped using real-time polymerase chain reaction (PCR) with 2.5 ng DNA as PCR template for allelic discrimination on an ABI7900HT (Life Technologies, Carlsbad, CA, USA). Genotype calls were obtained using SDS 2.3 software (Life Technologies, Carlsbad, CA, USA) with manual inspection and curation of fluorescence intensity plots. The call rate was >90%. The Hardy-Weinberg equilibrium (HWE) was calculated using an online calculator (chi-2 = 3.42, df = 2, *p* = 0.06).

### Statistical analysis

Subjects were categorized into sex-specific quartiles of leukocyte concentrations, i.e., four groups with the same proportion of men and women in each group. One-way analysis of variance (ANOVA) and logistic regression was used to assess cross-sectional relationships of leukocyte count to diabetes risk factors. A general linear model was used to adjust glucose and HbA_1c_ for potential confounding factors, in categories of the R262W polymorphism. Cox proportional hazards regression was used to examine hazard ratios (HR) with 95% confidence interval (CI) for incidence of diabetes by total and differential leukocyte counts (neutrophils, lymphocytes, and mixed cells) using the lowest quartile as the reference category. Time axis was follow-up time until death, emigration, incident diabetes or end of follow-up. The results were adjusted for age, sex, BMI and family history of diabetes in the basic model [17, 29]. Secondly, we also adjusted for waist circumference, systolic blood pressure, using of blood pressure- or lipid-lowering medication, history of cardiovascular disease, smoking habits, leisure physical activity, educational level and marital status. CRP was added to the covariates in an additional analysis in the MDC-CV sub-cohort. Possible interaction between leukocyte count and risk factors for diabetes was explored by introducing interaction terms in the fully adjusted multivariate model. The Kaplan-Meier curve was used to demonstrate incidence of diabetes in relation to total leukocyte count during the follow-up. The association between leukocyte count, fasting blood glucose and HbA1c, respectively, was analyzed using general linear model adjusted for age, sex and BMI. Model discrimination was estimated with Harrell’s C-statistics [30].

Sensitivity analyses were performed after excluding individuals having a cold or lung infection within two weeks before the baseline examination. We also explored the effect of non-steroid anti-inflammatory drug (NSAID) medication on the relationships between leukocytes and diabetes.

**Fig 1:**
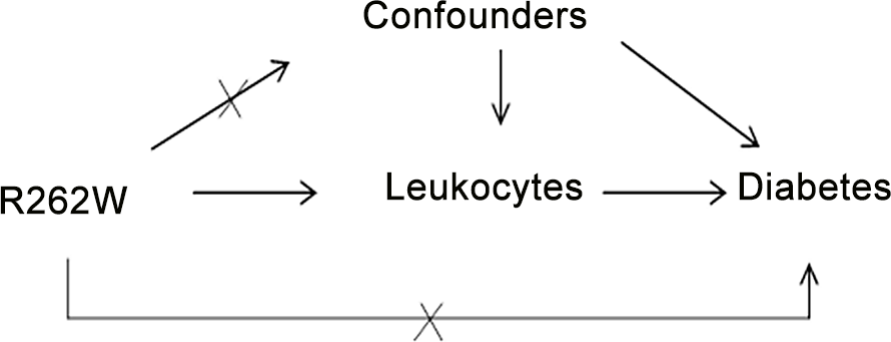
The Mendelian randomization path diagram. The relationship between the instrumental variable R262W, leukocytes (exposure variable), confounding factors and the outcome diabetes.

A Mendelian randomization analysis path diagram was presented in Fig 1. R262W was used as an instrumental variable for leukocyte count to study incidence of diabetes. Mendelian randomization analysis was performed with two-stage least squares regression (2SLS) using the ivreg2 command in STATA [31]. The genetic instrument was validated for association with total and differential leukocyte counts using linear or logistic regression models. A power analysis was performed for the association between R262W and incidence of diabetes. With α = 0.05, there was 64% power to detect a significant relationship, assuming a HR of 1.25 per standard deviation increment of leukocytes, i.e., the age and sex-adjusted HR in the present cohort.

All analyses were performed using IBM SPSS statistics (version 20; IBM Svenska AB, Stockholm, Sweden) and STATA12 (Stata Corp, College Station, TX, USA).

## Results

### Baseline Characteristics

The mean total leukocyte count was 6.37±1.68 x10^9^ /L, and the proportion of neutrophils, lymphocytes, and mixed cells were 61%, 31% and 8%, respectively. The relationships between sex-specific quartiles of total leukocyte count and risk factors for diabetes are presented for all subjects in Table 1, and separately for men and women in S1 Table and S2 Table. Increased leukocyte count was associated with age, BMI, waist circumference, systolic blood pressure, antihypertensive- or lipid-lowering medication, prevalent cardiovascular disease, smoking habits, low physical activity, education level and being married. In addition, HbA_1c_, glucose and insulin were positively and significantly associated with leukocyte count (p<0.001) in the subgroup, Table 1.

**Table 1:** Quartiles of total leukocytes and diabetes mellitus risk factors of participants in MDC (n = 26 667) and MDC-CV (n = 5 473).

	**MDC** (N = 26 667)				
**Sex-specific quartiles**	**Q1**	**Q2**	**Q3**	**Q4**	*p*
N (men/women)	2575/4246	2548/4097	2712/3790	2529/4170	
Leukocyte count, Men median (P_25_—P_75_), (10^9^/L)	4.70(4.20–5.20)	5.60(5.40–5.90)	6.60(6.40–6.90)	8.30(7.60–9.10)	
Leukocyte count, Women median (P_25_—P_75_) (10^9^/L)	4.70(4.0–5.00)	5.70(5.40–5.90)	6.70(6.50–7.00)	8.30(7.70–9.10)	
***Sociodemographic variables***					
Age (years)	57.6±7.0	58.4±7.7	58.4±7.7	57.8±8.0	<0.001
Married (%)	68.9	66.3	66.1	59.8	<0.001
Low education (%)	37.8	41.0	42.3	45.6	<0.001
***Anthropometric measurements***					
Waist circumference (cm)	81.6±15.3	83.5±12.6	85.0±16.0	85.0±13.2	<0.001
BMI (kg/m^2^)	25.0±3.5	25.7±3.8	25.9±4.0	26.0±4.2	<0.001
***Medical history variables***					
Family history of diabetes, (%)	2.0	2.0	1.8	1.7	0.226
Prevalent cardiovascular disease (%)	1.7	2.4	3.2	3.6	<0.001
Systolic blood pressure (mmHg)	138±19	141±20	142±20	143±20	<0.001
Antihypertensive medication (%)	12.2	15.8	18.4	21.3	<0.001
Lipid-lowing medication (%)	2.1	2.9	3.1	3.2	<0.001
***Lifestyle variables***					
Current smoker (%)	13.1	19.6	30.0	51.2	<0.001
Low physical activity (%)	20.8	22.5	24.3	28.5	<0.001
	**MDC-CV Subcohort** (n = 5 473)				
N (men/women)	508/768	643/883	508/762	586/815	
Leukocyte count, median, (men/women) (10^9^/L)	4.30/4.30	5.40/5.40	6.40/6.40	7.90/7.80	
Glucose (mmol/L) (n = 5082)	4.87±0.55	5.01±0.85	5.02±0.71	5.07±0.80	<0.001
HbA1c (%) (n = 5082)	4.70±0.40	4.79±0.49	4.84±0.51	4.97±0.55	<0.001
Insulin* (mIU/l) (n = 4912)	6.00(4.00–8.00)	7.00(4.00–9.00)	7.00(5.00–10.00)	7.00(4.75–10.00)	<0.001
CRP* (mg/L) (n = 4904)	0.90(0.50–1.80)	1.30(0.70–2.50)	1.50(0.80–3.10)	2.20(1.00–4.30)	<0.001

All values are mean±SD, unless otherwise stated.

*insulin, CRP is presented as median and interquartile limits, due to skewed distribution. *P* value for log-transform value.

MDC, Malmö Diet and Cancer.

### Incidence of diabetes in relation to total and differential leukocyte counts

**Fig 2:**
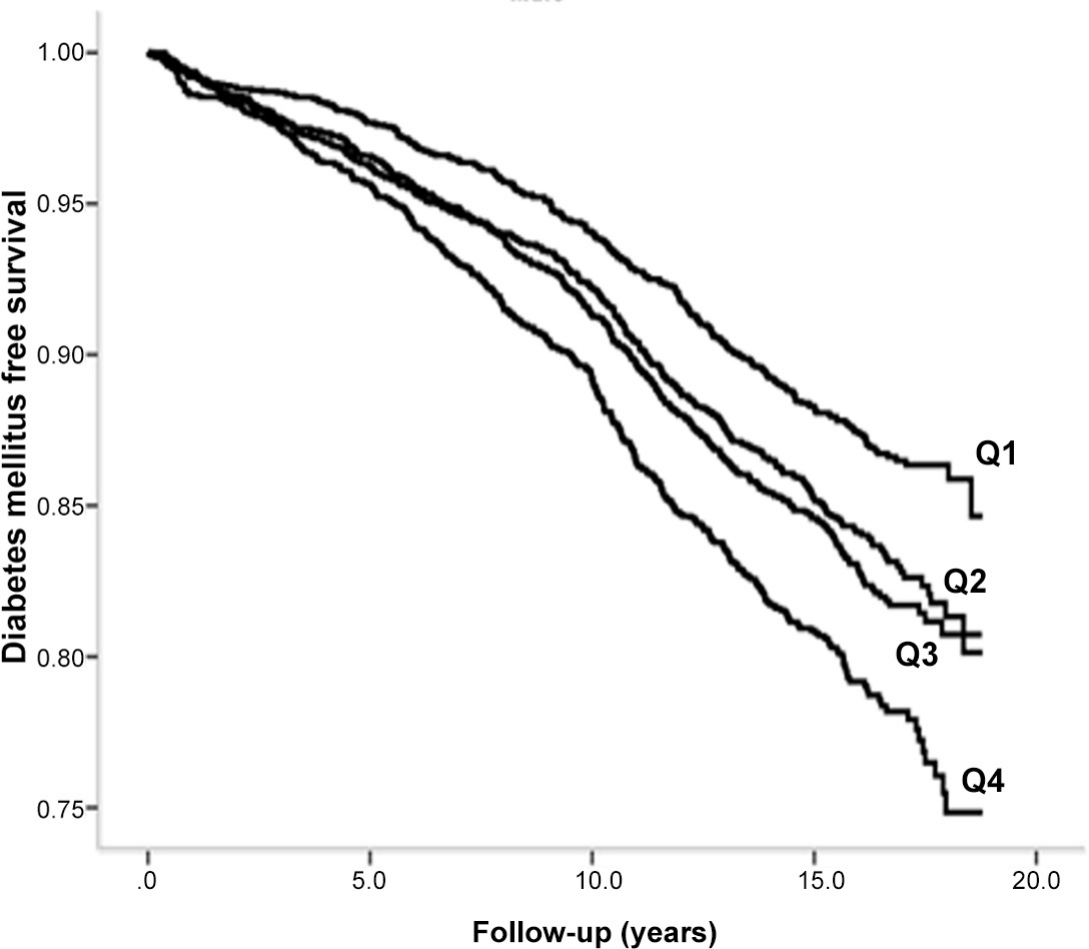
Diabetes mellitus free survival in relation to quartiles of total leukocytes in men.

**Fig 3:**
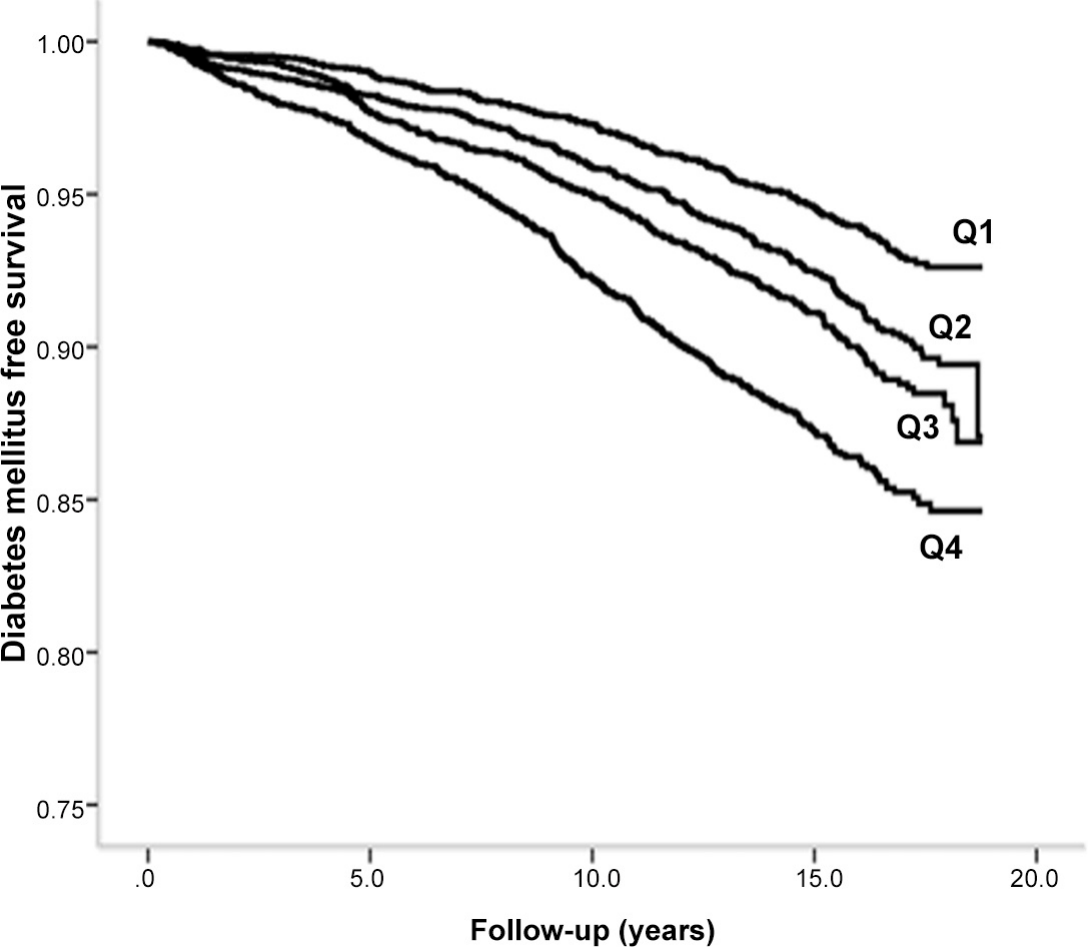
Diabetes mellitus free survival in relation to quartiles of total leukocytes in women.

During a mean follow-up of 14 years, 2 946 subjects (1 521 men and 1 425 women, 7.87 per 1000 person-years) developed diabetes. Kaplan-Meier curves of diabetes free survival in relation to sex-specific quartiles of total leukocyte count is shown in Figs 2 and 3. Incidence of diabetes was significantly associated with total and differential leukocyte counts in the basic model 1, Table 2. After adjustment for potential confounding factors, the association remained significant for total leukocyte (4^th^ vs 1^st^ quartiles HR: 95% CI; 1.37; 1.22–1.53), neutrophil (1.33; 95% CI: 1.19–1.49) and lymphocyte (1.29; 95% CI: 1.15–1.44) counts, but not for mixed cells (1.04; 95% CI: 0.94–1.15, not shown in table). Male sex, family history of diabetes, high BMI and waist circumference, high systolic blood pressure, use of antihypertensive and lipid-lowering medications, current smoking and low education level were all significantly associated with incidence of diabetes in the final multivariate model. No significant interaction was observed between total leukocyte count and other risk factors for diabetes. Use of non-steroid anti-inflammatory drugs (NSAID) (n = 822) at baseline was added to the multivariable adjusted model in a sensitivity analysis, but the results were essentially unchanged. NSAID was not a significant risk factor for diabetes in the model.

**Table 2:** Incidence of diabetes mellitus in relation to sex-specific quartiles of total and differential leukocyte counts in MDC cohort (N = 26 667).

	MDC (N = 26 667)				
Sex-specific quartiles	Q1	Q2	Q3	Q4	*p* for trend
Leukocyte count					
Incident diabetes, (men/women) (n/n)	305/249	369/324	405/346	442/506	
All^1^	1.00	1.17(1.05–1.31)	1.26(1.13–1.41)	1.72(1.54–1.91)	<0.001
All ^2^	1.00	1.08(0.97–1.21)	1.12(0.99–1.25)	1.37(1.22–1.53)	<0.001
Men ^1^	1.00	1.11(0.95–1.29)	1.17(1.01–1.36)	1.54(1.33–1.79)	<0.001
Men^2^	1.00	1.03(0.88–1.20)	1.05(0.90–1.23)	1.26(1.08–1.47)	0.003
Women^1^	1.00	1.24(1.05–1.46)	1.39(1.18–1.64)	1.97(1.69–2.30)	<0.001
Women^2^	1.00	1.14(0.97–1.35)	1.21(1.02–1.42)	1.53(1.30–1.79)	<0.001
Neutrophils					
Incident diabetes, (men/women) (n/n)	303/233	398/345	370/373	450/474	
All ^1^	1.00	1.22(1.09–1.36)	1.24(1.11–1.38)	1.65(1.48–1.83)	<0.001
All ^2^	1.00	1.15(1.02–1.28)	1.10(0.98–1.23)	1.33(1.19–1.49)	<0.001
Men^1^	1.00	1.17(1.01–1.36)	1.13(0.97–1.32)	1.54(1.33–1.78)	<0.001
Men^2^	1.00	1.09(0.93–1.26)	1.01(0.87–1.18)	1.26(1.09–1.47)	0.008
Women^1^	1.00	1.29(1.09–1.52)	1.40(1.19–1.65)	1.85(1.58–2.16)	<0.001
Women^2^	1.00	1.22(1.03–1.44)	1.23(1.04–1.45)	1.46(1.24–1.73)	<0.001
Lymphocytes					
Incident diabetes, (men/women) (n/n)	284/233	319/370	442/353	476/469	
All ^1^	1.00	1.10(0.98–1.24)	1.22(1.09–1.37)	1.46(1.31–1.63)	<0.001
All ^2^	1.00	1.06(0.95–1.19)	1.13(1.01–1.26)	1.29(1.15–1.44)	<0.001
Men^1^	1.00	1.02(0.87–1.19)	1.12(0.96–1.30)	1.28(1.10–1.48)	<0.001
Men^2^	1.00	0.99(0.85–1.17)	1.06(0.91–1.23)	1.16(1.00–1.35)	0.026
Women^1^	1.00	1.20(1.02–1.41)	1.32(1.12–1.56)	1.70(1.45–1.99)	<0.001
Women^2^	1.00	1.13(0.96–1.34)	1.20(1.02–1.42)	1.43(1.22–1.69)	<0.001

All values are Hazard ratio (HR) (95%CI), unless otherwise stated.

^1^HR adjusted for age, sex, BMI and family history of diabetes.

^2^HR (^1^) plus adjusted for waist, systolic blood pressure, blood pressure-lowering medication, lipid-lowering medication, prevalent cardiovascular disease, smoking habits, physical activities, marital status and education level. Cl, confidence interval. MDC, Malmö Diet and Cancer.

We also performed a sensitivity analysis after excluding individuals who reported having a cold or lung infection within two weeks before the baseline examination (n = 20 906). After adjustment for potential confounding factors, the association remained significant for total leukocyte (4th vs 1st quartiles HR: 1.39; 95% CI; 1.22–1.58), neutrophil (1.37; 95% CI: 1.21–1.56) and lymphocyte (1.32; 95% CI: 1.16–1.50) counts, while mixed cells remained non-significant (1.03; 95% CI: 0.92–1.16).

In the MDC-CV subcohort, 736 subjects developed diabetes during the follow-up. The HR (95% CI) for total leukocyte count (4^th^ vs 1^st^ quartile) was 1.95 (1.55–2.46, P for trend <0.001) in the basic model, S3 Table. The HR was reduced to 1.53 (1.20–1.95; *P* for trend 0.004) taking possible confounders into account and remained significant also after adding CRP into the model (HR: 1.37; 1.05–1.77; *P* for trend 0.044). Among the leukocyte subpopulations, only neutrophils remained significantly associated with incidence of diabetes after adjustment for CRP (HR: 1.39; 1.08–1.78, P for trend 0.022), S3 Table.

The C-statistics value for a model with age, sex and BMI was 0.717 (0.708–0.726) and increased to 0.722 (0.713–0.731) when leukocyte count was added to the model. Leukocyte count significantly improved the discriminatory value (C-statistic) for incidence of diabetes with 0.005 (0.002–0.008) (p<0.001).

### Polymorphism in the *SH2B3* gene

The association between the R262W polymorphism and leukocyte count is shown in Table 3. The frequency of the minor allele (T) of R262W was 48%. The T allele was strongly associated with increased leukocytes (0.11x10^9^ cells/l per T allele, *p* = 1.14 x10^-12^, F statistics = 50.6), lymphocytes (p = 4.3 x10^-16^), neutrophils (p = 8.0 x10^-6^) and mixed cells (p = 3.0 x10^-6^). There was no association between the R262W polymorphism and BMI (p = 0.154), waist (p = 0.495), systolic blood pressure (p = 0.231) and use of lipid-lowering medication (p = 0.723).

**Table 3:** Baseline characteristics of the study population divided by alleles (CC, CT, TT) of R262W (N = 24 489).

	CC	CT	TT	P for ANOVA
N (%)	6772 (27.7)	12074(49.3)	5643(23.0)	
Leukocyte count, (10^9^/L)	6.3±1.7	6.4±2.3	6.5±1.9	<0.001
Sex, men, %	38.3	38.6	39.1	0.680
Age (years)	58.3±7.7	58.0±7.7	58.1±7.6	0.049
BMI (kg/m^2^)	25.7±3.9	25.6±3.9	25.7±4.0	0.154
Waist (cm)	83.7±12.8	83.6±12.7	83.8±12.9	0.495
Incident diabetes n (per 1000 person-year)	762 (8.0)	1309 (7.7)	644 (8.2)	0.465
Glucose (mmol L^−1^) (n = 4964)	5.02±0.81	4.99±0.71	4.99±0.74	0.562
HbA_1c_ (%) (n = 4966)	4.84±0.54	4.81±0.48	4.81±0.47	0.219
Insulin* (mIU/l) (n = 4798)	6.0(4.0–9.0)	6.0(4.0–9.0)	6.0(4.0–9.0)	0.067

Values are as means ± standard deviations or %. BMI: body mass index.

*insulin is presented as median and interquartile limits due to skewed distribution.

We found no statistically significant association between the R262W polymorphism and incidence of diabetes, fasting blood glucose or HbA_1c_, Table 3. These relationships remained non-significant after full adjustment for potential confounding factors in multivariable Cox regression (diabetes) or general linear models (glucose, HbA_1c_) (all p>0.288). For glucose, the IV estimator was -0.210 (95% CI: -0.723–0.302) (*p* = 0.421); for HbA_1c_, the IV estimator was -0.201 (-0.558–0.157) (*p* = 0.272). For diabetes, the IV estimator was -0.005 (-0.047–0.057)(*p* = 0.837).

## Discussion

The present study showed a graded association between concentrations of leukocytes, neutrophils and lymphocytes and risk of developing diabetes among middle-aged subjects, taking many potential confounding factors into account. The results confirm that leukocyte count is a risk factor for incidence of diabetes. However, a missense polymorphism in the *SH2B3* gene, strongly associated with leukocyte count, was not related to glucose, HbA_1c_ or incidence of diabetes. This suggests that the relationship between leukocytes and diabetes might not be causal.

Previous studies have reported that various inflammation markers, e.g., interleukin-6, tumor necrosis factor α (TNFα) and CRP are associated with diabetes [1, 2, 32] It is believed that TNFα contributes to diabetes through its interaction with insulin signaling pathways and beta-cell function [33]. Since human granulocytes secrete TNFα, this could be a possible link between leukocyte count and diabetes [34]. A polymorphism in the IL-6 gene has been associated with total and differential white blood cell counts [35]. Since IL-6 is produced by human mononuclear cells, this suggests that IL-6 might be a common link between leukocyte count and diabetes [36]. Hence, the relationship between leukocytes and diabetes could be related to the actions of various pro-inflammatory cytokines.

Observational studies could be limited by unmeasured confounding. However, as the alleles are randomly assigned at meiosis and fixed through the lifetime, genetic association studies are usually not subject to confounding. The R262W polymorphism, which is a non-synonymous SNP located in exon 3 of *SH2B3*, leads to an amino acid change in the pleckstrin homology domain. *SH2B3* regulates cytokine receptor-mediated signaling implicated in leukocyte activation [37, 38]. The R262W polymorphism was strongly associated with leukocyte count (F statistics value = 50.6), but we did not find any association between R262W and diabetes, glucose or HbA1c. The results indicate that the relationship between leukocytes and diabetes might not be causal. However, it should be acknowledged that the leukocyte population is highly complex with many different subpopulations [10]. It remains possible that specific populations of leukocytes could be causally associated with diabetes. In addition, even though the number of participants was high and the SNP can be considered a fairly strong instrument, it is still possible that the statistical power was too small in this study. The minor allele of the R262W polymorphism has been previously associated with increased risk of several autoimmune diseases including type 1 diabetes [39], multiple sclerosis [40], blood pressure [41] and MI [9]. Some of these disorders could increase the probability that diabetes is detected and that antihypertensive treatments are prescribed that could increase blood glucose levels. It is not possible to exclude the possibility of pleiotropic effects. However, a potential relationship between the minor allele and increased risk of type 1 diabetes, MI and hypertension should increase the risk of diabetes and cannot explain the negative results in this study.

### Strength and limitations

The strength of the study was the large numbers of subjects and events during a long follow-up period. A limitation of the present study is lack of information on type of diabetes. Participants were 45–73 years old and non-diabetic at the baseline examination. It can be assumed that almost all incident cases developed type 2 diabetes, since type 1 diabetes usually has an early onset, and were excluded from analyses as prevalent cases [42]. New cases of diabetes were identified from several independent sources. The registers of out- and in-patients cover all hospital visits in the country and the pharmaceutical register covers all filled prescriptions from all pharmacies in Sweden since 2005. The HbA_1c_ register covers the population in the city of Malmö. The relationship between leukocytes and diabetes was largely the same for each of the data sources. Diabetes can go undetected for several years and individuals that do not seek medical care will be missed. However, the coverage of the registers is very good and we have no reason to question the case validity. Lack of information about change of exposure during the follow-up (e.g. weight change, quitting smoking, new medication, etc.) was another possible limitation. A further limitation is the potential pleiotropy for the *SH2B3* missense variant, which is associated with many traits. Although this would be more relevant in the context of a positive finding, it could be hypothesized that a pleiotropic effect could attenuate any association with diabetes. So-called canalization, i.e., compensatory mechanisms that counterbalance the effects of the genetic instrument, is another possibility that hypothetically could explain the negative results for the R262W polymorphism.

Although the leukocyte count significantly increased the model discrimination in terms of C-statistics, it is uncertain whether measurements of leukocytes could improve prediction of future diabetes in clinical practice. However, further studies are needed to confirm this. In conclusion, increased leukocyte counts are associated with incidence of diabetes. However, the negative findings for the R262W polymorphism suggest that the associations may not be causal, although limitations in statistical power and balancing pleiotropic effects cannot be excluded. Further studies are needed for replication of this finding in other cohorts.

## Supporting Information

S1 Table Quartiles of total leukocytes and diabetes mellitus risk factors of participants in MDC and MDC-CV in men. (DOC)

S2 Table Quartiles of total leukocytes and diabetes mellitus risk factors of participants in MDC and MDC-CV in women. (DOC)

S3 Table Incidence of diabetes mellitus in relation to sex-specific quartiles of total leukocytes and neutrophils in MDC-CV cohort (n = 5 473). (DOC)
