# Supplementary material for: Total and Differential Leukocyte Counts in Relation to Incidence of Diabetes Mellitus: A Prospective Population-Based Cohort Study
Source: PLoS One. 2016 Feb 18;11(2):e0148963. doi: 10.1371/journal.pone.0148963 (PMC4758613; doi:10.1371/journal.pone.0148963)
Supplement: S1 Table — (DOC) [file pone.0148963.s001.doc]

S1 Table. Quartiles of total leukocytes and diabetes mellitus risk factors of participants in MDC and MDC-CV in men.

|  | **MDC** | | | | | | |
| --- | --- | --- | --- | --- | --- | --- | --- |
| **Sex-specific quartiles of Leukocyte count** | **Q1** | **Q2** | **Q3** | | **Q4** | | *p* |
| N | 2575 | 2548 | 2712 | | 2529 | |  |
| Leukocyte count , median (10^9^/L) | 4.70 | 5.60 | 6.60 | | 8.30 | |  |
| ***Sociodemographic variables*** |  |  |  | |  | |  |
| Age (years) | 57.9±6.8 | 59.5±7.0 | 59.6±7.1 | | 59.4±7.2 | | <0.001 |
| Married (%) | 74.7 | 74.1 | 74.4 | | 67.7 | | <0.001 |
| Low education (%) | 40.0 | 45.8 | 46.9 | | 50.7 | | <0.001 |
| ***Anthropometric measurements*** |  |  |  | |  | |  |
| Waist circumference (cm) | 91.7±9.1 | 93.5±9.6 | 94.2±10.0 | | 94.3±10.7 | | <0.001 |
| BMI (kg/m^2^) | 25.7±3.1 | 26.3±3.4 | 26.4±3.5 | | 26.3±3.7 | | <0.001 |
| ***Medical history variables*** |  |  |  | |  | |  |
| Family history of diabetes, (%) | 1.6 | 1.7 | 1.6 | | 1.7 | | 0.802 |
| Prevalent cardiovascular disease (%) | 3.1 | 4.7 | 5.8 | | 6.8 | | <0.001 |
| Systolic blood pressure (mmHg) | 140±18 | 144±19 | 144±19 | | 146±20 | | <0.001 |
| Antihypertensive medication (%) | 13.5 | 18.5 | 21.1 | | 23.6 | | <0.001 |
| Lipid-lowing medication (%) | 3.4 | 4.5 | 4.3 | | 5.3 | | 0.010 |
| ***Lifestyle variables*** |  |  |  | |  | |  |
| Current smoker (%) | 13.0 | 19.2 | 29.4 | | 53.8 | | <0.001 |
| Low physical activity (%) | 20.1 | 23.6 | 24.0 | | 28.0 | | <0.001 |
|  | **MDC-CV Subcohort** | | | | | | |
| N | 508 | 643 | | 508 | | 586 |  |
| Leukocyte count, median, (10^9^/L) | 4.30 | 5.40 | | 6.40 | | 7.90 |  |
| Glucose (mmol/L) | 5.06±0.64 | 5.20±1.04 | | 5.19±0.76 | | 5.22±0.90 | 0.013 |
| HbA1c (%) | 4.66±0.42 | 4.77±0.55 | | 4.83±0.57 | | 4.96±0.61 | <0.001 |
| Insulin*, ( mIU/l) | 6.00(4.00-9.00) | 7.00(5.00-10.00) | | 7.00(5.00-10.00) | | 7.00(5.00-11.00) | 0.013 |
| CRP*, (mg/L) | 0.90(0.50-1.70) | 1.30(0.70-2.60) | | 1.50(0.80-3.00) | | 2.60(1.20-4.55) | <0.001 |

All values are mean±SD, unless otherwise stated.

*insulin, CRP is presented as median (interquartile limits) due to skewed distribution. *P* value for log-transform value.
