# Supplementary material for: Total and Differential Leukocyte Counts in Relation to Incidence of Diabetes Mellitus: A Prospective Population-Based Cohort Study
Source: PLoS One. 2016 Feb 18;11(2):e0148963. doi: 10.1371/journal.pone.0148963 (PMC4758613; doi:10.1371/journal.pone.0148963)
Supplement: S2 Table — (DOC) [file pone.0148963.s002.doc]

S2 Table. Quartiles of total leukocytes and diabetes mellitus risk factors of participants in MDC and MDC-CV in women.

|  | **MDC** | | | | |
| --- | --- | --- | --- | --- | --- |
| **Sex-specific quartiles of Leukocyte count** | **Q1** | **Q2** | **Q3** | **Q4** | *p* |
| N | 4246 | 4097 | 3790 | 4170 |  |
| Leukocyte count , median (10^9^/L) | 4.70 | 5.70 | 6.70 | 8.30 |  |
| ***Sociodemographic variables*** |  |  |  |  |  |
| Age (years) | 57.4±7.1 | 57.7±8.0 | 57.5±8.0 | 56.8±8.3 | <0.001 |
| Married (%) | 65.5 | 61.5 | 60.1 | 55.0 | <0.001 |
| Low education (%) | 36.4 | 38.0 | 39.0 | 42.5 | <0.001 |
| ***Anthropometric measurements*** |  |  |  |  |  |
| Waist circumference (cm) | 75.3±8.9 | 77.3±9.9 | 78.3±10.5 | 79.3±11.2 | <0.001 |
| BMI (kg/m^2^) | 24.6±3.6 | 25.3±4.0 | 25.6±4.3 | 25.8±4.5 | <0.001 |
| ***Medical history variables*** |  |  |  |  |  |
| Family history of diabetes, (%) | 2.2 | 2.2 | 1.9 | 1.8 | 0.497 |
| Prevalent cardiovascular disease (%) | 0.8 | 0.9 | 1.3 | 1.6 | 0.003 |
| Systolic blood pressure (mmHg) | 137±19 | 139±20 | 139±20 | 140±20 | <0.001 |
| Antihypertensive medication (%) | 11.4 | 14.1 | 16.5 | 19.9 | <0.001 |
| Lipid-lowing medication (%) | 1.4 | 1.9 | 2.2 | 1.9 | 0.043 |
| ***Lifestyle variables*** |  |  |  |  |  |
| Current smoker (%) | 13.1 | 19.8 | 30.4 | 49.6 | <0.001 |
| Low physical activity (%) | 21.3 | 21.9 | 24.5 | 28.8 | <0.001 |
|  | **MDC-CV Subcohort** | | | | |
| N | 768 | 883 | 762 | 815 |  |
| Leukocyte count, median,(10^9^/L) | 4.30 | 5.40 | 6.40 | 7.80 |  |
| Glucose (mmol/L) | 4.76±0.45 | 4.89±0.65 | 4.90±0.65 | 4.97±0.71 | <0.001 |
| HbA1c (%) | 4.72±0.39 | 4.80±0.45 | 4.85±0.47 | 4.92±0.51 | <0.001 |
| Insulin*, ( mIU/l) | 5.00(3.00-8.00) | 6.00(4.00-9.00) | 6.00(4.00-9.00) | 7.00(4.00-9.00) | <0.001 |
| CRP*, (mg/L) | 1.0(0.50-1.90) | 1.3(0.70-2.50) | 1.6(0.80-3.10) | 2.05(0.90-4.30) | <0.001 |

All values are mean±SD, unless otherwise stated.

*insulin, CRP is presented as median (interquartile limits) due to skewed distribution. *P* value for log-transform value.
