# Supplementary material for: Total and Differential Leukocyte Counts in Relation to Incidence of Diabetes Mellitus: A Prospective Population-Based Cohort Study
Source: PLoS One. 2016 Feb 18;11(2):e0148963. doi: 10.1371/journal.pone.0148963 (PMC4758613; doi:10.1371/journal.pone.0148963)
Supplement: S3 Table — (DOC) [file pone.0148963.s003.doc]

S3 Table. Incidence of diabetes mellitus in relation to sex-specific quartiles of total leukocytes and neutrophils in MDC-CV cohort (n=5 473).

|  | **MDC-CV**(n=5 473) | | | | |
| --- | --- | --- | --- | --- | --- |
| **Sex-specific quartiles** | **Q1** | **Q2** | **Q3** | **Q4** | *p* for trend |
| Leukocyte count |  |  |  |  |  |
| Incident diabetes, (men/women) (n/n) | 62/41 | 107/108 | 76/108 | 119/115 |  |
| All^1^ | 1.00 | 1.58(1.24-2.00) | 1.59(1.25-2.03) | 1.95(1.55-2.46) | <0.001 |
| All ^2^ | 1.00 | 1.44(1.13-1.82) | 1.42(1.13-1.82) | 1.53(1.20-1.95) | 0.004 |
| All ^3^ | 1.00 | 1.34(1.04-1.72) | 1.37(1.07-1.77) | 1.37(1.05-1.77) | 0.044 |
| Men^1^ | 1.00 | 1.18(0.86-1.62) | 1.09(0.78-1.53) | 1.63(1.20-2.22) | 0.002 |
| Men ^2^ | 1.00 | 1.10(0.80-1.51) | 0.94(0.67-1.33) | 1.25(0.90-1.74) | 0.251 |
| Men^3^ | 1.00 | 1.03(0.74-1.43) | 0.83(0.58-1.19) | 1.11(0.79-1.57) | 0.741 |
| Women^1^ | 1.00 | 2.15(1.50-3.09) | 2.38(1.66-3.42) | 2.47(1.73-3.54) | <0.001 |
| Women^2^ | 1.00 | 1.94(1.35-2.79) | 2.22(1.54-3.19) | 1.95(1.74-2.84) | 0.002 |
| Women^3^ | 1.00 | 2.00(1.36-2.94) | 2.23(1.51-3.29) | 1.82(1.21-2.71) | 0.021 |
| Neutrophils |  |  |  |  |  |
| Incident diabetes, (men/women) (n/n) | 68/55 | 98/76 | 89/123 | 109/118 |  |
| All ^1^ | 1.00 | 1.38(1.09-1.74) | 1.46(1.17-1.83) | 1.88(1.51-2.34) | <0.001 |
| All ^2^ | 1.00 | 1.30(1.03-1.64) | 1.30(1.04-1.63) | 1.52(1.21-1.92) | <0.001 |
| All ^3^ | 1.00 | 1.28(1.00-1.64) | 1.26(0.99-1.60) | 1.39(1.08-1.78) | 0.022 |
| Men^1^ | 1.00 | 1.28(0.94-1.75) | 1.02(0.75-1.41) | 1.63(1.21-2.22) | 0.009 |
| Men^2^ | 1.00 | 1.20(0.88-1.64) | 0.90(0.65-1.24) | 1.27(0.92-1.75) | 0.953 |
| Men^3^ | 1.00 | 1.12(0.81-1.55) | 0.79(0.56-1.11) | 1.11(0.79-1.57) | 0.400 |
| Women^1^ | 1.00 | 1.49(1.05-2.11) | 2.03(1.48-2.80) | 2.23(1.62-3.08) | <0.001 |
| Women^2^ | 1.00 | 1.41(0.99-2.00) | 1.86(1.35-2.57) | 1.88(1.35-2.62) | <0.001 |
| Women^3^ | 1.00 | 1.49(1.03-2.16) | 2.03(1.44-2.87) | 1.79(1.25-2.57) | <0.001 |

All values are Hazard ratio (HR) (95%CI), unless otherwise stated.

^1^HR adjusted for age, sex, BMI and family history of diabetes.

^2^HR (^1^) and adjusted for waist, systolic blood pressure, blood pressure-lowering medication, lipid-lowering medication, prevalent cardiovascular disease, smoking habits, physical activities, marital status, and education level.

^3^HR (^2^) and adjusted for CRP.

Cl, confidence interval. MDC-CV Malmö Diet and Cancer-cardiovascular cohort.
